# Mutant PIK3CA Induces EMT in a Cell Type Specific Manner

**DOI:** 10.1371/journal.pone.0167064

**Published:** 2016-12-12

**Authors:** Divya Bhagirath, Xiangshan Zhao, Sameer Mirza, William W. West, Hamid Band, Vimla Band

**Affiliations:** 1 Department of Genetics, Cell Biology and Anatomy, University of Nebraska Medical Center, Omaha, Nebraska, United States of America; 2 Pathology & Microbiology, College of Medicine, University of Nebraska Medical Center, Omaha, Nebraska, United States of America; 3 Eppley Institute for Cancer and Allied Diseases, University of Nebraska Medical Center, Omaha, Nebraska, United States of America; 4 Fred & Pamela Buffett Cancer Center, University of Nebraska Medical Center, Omaha, Nebraska, United States of America; University of California Davis, UNITED STATES

## Abstract

Breast cancer is characterized into different molecular subtypes, and each subtype is characterized by differential gene expression that are associated with distinct survival outcomes in patients. PIK3CA mutations are commonly associated with most breast cancer subtypes. More recently PIK3CA mutations have been shown to induce tumor heterogeneity and are associated with activation of EGFR-signaling and reduced relapse free survival in basal subtype of breast cancer. Thus, understanding what determines PIK3CA induced heterogeneity and oncogenesis, is an important area of investigation. In this study, we assessed the effect of mutant PIK3CA together with mutant Ras plus mutant p53 on oncogenic behavior of two distinct stem/progenitor breast cell lines, designated as K5+/K19- and K5+/K19+. Constructs were ectopically overexpressed in K5+/K19- and K5+/K19+ stem/progenitor cells, followed by various *in-vitro* and *in-vivo* analyses. Oncogene combination m-Ras/m-p53/m-PIK3CA efficiently transformed both K5+/K19- and K5+/K19+ cell lines *in-vitro*, as assessed by anchorage-independent soft agar colony formation assay. Significantly, while this oncogene combination induced a complete epithelial-to-mesenchymal transition (EMT) in K5+/K19- cell line, mostly epithelial phenotype with minor EMT component was seen in K5+/K19+ cell line. However, both K5+/K19- and K5+/K19+ transformed cells exhibited increased invasion and migration abilities. Analyses of CD44 and CD24 expression showed both cell lines had tumor-initiating CD44^+^/CD24^low^ cell population, however transformed K5+/K19- cells had more proportion of these cells. Significantly, both cell types exhibited *in-vivo* tumorigenesis, and maintained their EMT and epithelial nature *in-vivo* in mice tumors. Notably, while both cell types exhibited increase in tumor-initiating cell population, differential EMT phenotype was observed in these cell lines. These results suggest that EMT is a cell type dependent phenomenon and does not dictate oncogenesis.

## Introduction

Breast cancer is a heterogeneous disease and is classified into different molecular subtypes, namely- luminal-like, ErbB2 over-expressing, basal-like and claudin-low [1–3]. Comprehensive analysis of large cohort of patient derived breast tumors have led to identification of various subtype specific gene alterations [4–6]. Recurrent gene changes, such as mutations in PIK3CA, TP53, MAP3K1, RUNX1, gene amplification/over-expression of ErbB2, loss of tumor suppressor PTEN, and RB, and their association with different breast cancer subtypes, signifies an important gene alteration and subtype relationship [4, 5, 7]. Furthermore, each subtype is associated with distinct survival outcomes, emphasizing an important role of these oncogenes in disease pathogenesis [2, 3]. PIK3CA mutation is found to be commonly associated with most breast tumors, including luminal-like, ErbB2-over-expressing and basal-like subtype [4]. Mutant PIK3CA in combination with mutant Ras has been shown to efficiently transform hMECS *in-vitro* [8, 9]. More recently, it has been demonstrated that induction of PIK3CA mutation in different cell lineages affects the phenotype of resulting mice tumors [10]. Furthermore, activation of EGFR signaling (up-regulated in basal subtype) in the presence of mutant PIK3CA has been shown to be associated with reduced relapse free survival [11]. Therefore, understanding the role of mutant PIK3CA in basal breast cancer (BC) subtype pathogenesis is of obvious significance.

We previously demonstrated that overexpression of oncogene combinations mRas/mp53/wtErbB2 or mRas/mp53/wtEGFR efficiently transformed two different basal subtypes of mammary stem/progenitor cell lines (probably representing different lineages in basal mammary epithelial cell hierarchy) designated as K5+/K19- and K5+/K19+ [12]. Both the transformed cell types gave rise to heterogeneous tumors when transplanted *in-vivo* and showed variations in incidence and latency for tumor and metastasis formation. K5+/K19- cells transformed by oncogene combination mRas/mp53/wtErbB2 generated primary tumors with shorter latency in comparison to K5+/K19- cells transformed by mRas/mp5/wtEGFR. Although, primary tumor onset was significantly delayed for mRas/mp5/wtEGFR transformed K5+/K19- cells, these cell lines exhibited similar latency for developing lung metastasis as that of K5+/K19- cells transformed by mRas/mp53/wtErbB2. We also observed that transformed K5+/K19+ cell type overall had a higher metastasis formation ability than transformed K5+/K19- cells [12]. Given, these significant differential effects of oncogenes and cell type on breast tumor pathogenesis, in the present study we investigated the effect of overexpression of mutant PIK3CA (H1047R) in combination with mRas (Q61L) and mp53(R249S) on oncogenesis of stem/progenitor K5+/K19- and K5+/K19+ cells.

We report that overexpression of oncogene combination mRas/mp53/mPIK3CA in both cell types induces complete transformation, as assessed by increased anchorage independence and increased invasion/migration *in-vitro*, increased levels of CD44+/CD24^low^ tumor-initiating population; and *in-vivo* tumors when orthotopically implanted into mammary glands of NOD/SCID gamma (NSG) mice. Significantly, however only K5+/K19- cells showed a clear EMT phenotype both *in-vitro* and *in-vivo*, while K5+/K19+ cells displayed mostly epithelial phenotype with minor EMT. Taken together, this study suggest EMT is cell type dependent rather than oncogene dependent and EMT does not necessarily correlate with *in vitro or in vivo* oncogenic behavior of cells.

## Materials and Methods

### Cell lines and retroviral/lentiviral infection

Mutant p53-R249S in pLENTI-6 (purchased from Addgene) along with Invitrogen packaging vector (ViraPowerTM Lentiviral Packaging MIX) were transfected into 293FT packaging cells. Lentiviral supernatants were collected after overnight incubation in fresh DMEM media. TSA54 packaging cells were transfected with retroviral constructs, mutant H-Ras Q61L in pBABE-hygro or mPIK3CA-H1047R in pMSCV-puro vector, together with PIK plasmid for packaging, and viral supernatants were collected (as mentioned above for lentiviral). K5+/K19- and K5+/K19+ stem/progenitor cell lines described previously [13], were infected with viral supernatants with different gene combinations followed by selection in DFCI-1 medium [14, 15] containing hygromycin (15 μg/ml) (for mutant H-Ras), blasticidine (15 μg/ml) (for mutant p53), puromycin (0.5 μg/ml) (for mPIK3CA).

### Antibodies

The following antibodies were used for western blotting, immunofluorescence, flow-cytometry and IHC: mouse anti-human p53 (DO-1) (sc-126), mouse anti-human α-smooth muscle actin (SMA) (sc-32251), mouse anti-human vimentin (sc-6260)—all were from Santa Cruz Biotechnology. Mouse anti-human Ras (610001), mouse anti-human MUC1 (550486), FITC conjugated anti-CD24 (555427), PE-conjugated anti-CD44 (555479) and Alexa-488 conjugated E-Cadherin (560061), all were from BD Bioscience. Rabbit anti-human vimentin (clone SP20, RM-9120-S0) was from Thermo Scientific. Rabbit anti-human K5 (PRB-160P) was from Covance. Rabbit anti-human PI3 Kinase p110 α (4255S) was from Cell Signaling.

### Anchorage independence growth assay

As described previously [12], briefly 40,000 cells suspended in DFCI-1 medium containing 0.3% agarose were seeded on top layer of 0.6% agarose as a bottom layer in 6-well plates. Each cell line was plated in triplicates.

### Flow cytometry analysis

1 million cells were incubated with FITC conjugated anti-CD24 and PE-conjugated anti-CD44 at 4°C for 45 min. Following incubation, the cells were washed three times with washing buffer (phosphate buffer saline with 0.2% fetal bovine serum) and were then subjected to fluorescence-activated cell sorter (FACS) analysis.

### Migration and invasion assay

20,000 cells were plated in trans-well chambers (BD Biosciences) and incubated at 37°C for 13 or 23 hrs. Cells in upper chamber were cleared using cotton swab, while the cells in the bottom chambers were fixed with HEMA fixative (#122-911A), followed by staining with HEMA solution I (#122-911B) and HEMA solution II (#122-911C) for 5 min. each. Stained cells were counted under the inverted microscope.

### *In-vitro* matrigel polarity assay

Protocol used for matrigel assay has been described previously [16, 17]. Briefly, 1000 cells suspended in DFCI-1 medium containing 2% matrigel were plated on glass coverslips with 100% reconstituted basement membrane (matrigel from BD Biosciences) in 24 well plate. Cells were cultured for 12 days with alternate day feeding.

### *In-vitro* tumorsphere formation assays

Protocol was similar as described earlier (12). Briefly 40,000 cells were plated in ultra-low attachment 6 well plates (Corning) in mammary epithelial growth medium (MEGM), as described previously by Dontu et al. Each cell line had 6 replicates. Cells were fed with fresh medium on alternate days. Tumorspheres were counted under the microscope after 3 weeks of plating. Tumorpheres were trypsinized and plated again for secondary or tertiarytumorsphere assays.

### Xenograft transplantation assays for primary tumor formation

6–8 weeks old immuno-deficient NSG mice (purchased from Jackson laboratories) were orthotopically injected with 1 million cells in DFCI-1 medium mixed with matrigel in 1:1 proportion [18] in the fourth and ninth (contralateral) mammary glands. Tumor formation was assessed by palpation in the area of injection every week until 6 months. After six months, mice with or without tumors were sacrificed by CO_2_ inhalation followed by cervical dislocation. Tumors were excised, fixed with 10% neutral buffered formalin, processed to prepare paraffin-embedded tumor blocks that were then sectioned for IHC. This study was approved by the Institutional Animal Care and Use Committee (IACUC) of University of Nebraska Medical Center, Omaha, NE (Protocol No. 10-086-12-FC).

### Immunohistochemistry

As described previously [12, 19], 4 μm sections were cut from the paraffin blocks and stained with indicated antibodies. Staining procedure was performed as per the manufacturer’s protocol (# K4007). For IHC staining, tissue sections were incubated with primary antibodies (anti-K5, anti-MUC1, anti-vimentin or anti-α-SMA) in a hydrated chamber, followed by incubation with HRP-tagged secondary IgG against the primary antibodies and DAB solution and subsequently processed for nuclear staining with hematoxylin and mounting of tissues. For double-immunofluorescence staining tumor sections were processed similarly and blocked with 10% goat serum for 1 hr, followed by staining with rabbit anti-vimentin, alexa 488 conjugated anti-E-cadherin or mouse anti- α-SMA antibodies. Goat anti-rabbit alexa 594 and goat anti-mouse alexa 488 conjugated secondary antibodies (Invitrogen) were used for staining. Sections were mounted with anti-fade mounting media and images were taken with fluorescence microscope (Zeiss axioplan 2 imaging microscope).

### Isolation and culture of primary tumor derived cells

Xenograft tumors were excised from NSG mice and then minced with sterilized scalpel in clean P-60 petri dishes. Minced tumors were cultured in either α-MEM or DFCI-1 medium for 1–2 weeks, following which the cells were trypsinized, and subjected to antibiotic (blasticidine, hygromycin and puromycin) selection to isolate human cells.

## Results

### Overexpression of mRas/mp53/mPIK3CA leads to oncogenic transformation of K5+/K19- and K5+/K19+ cells

**Fig 1:**
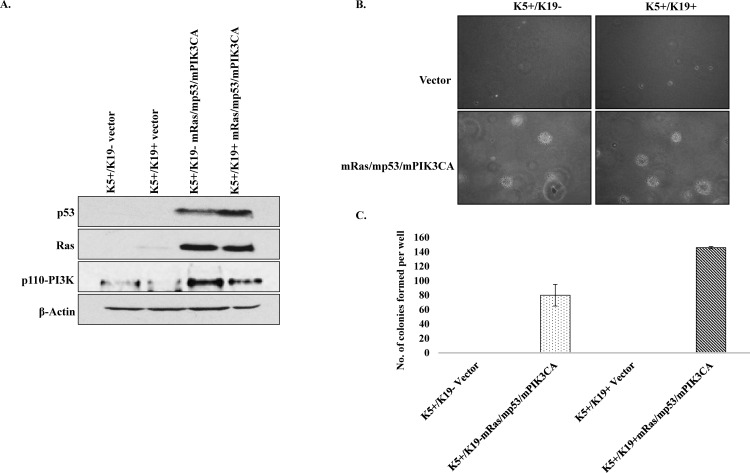
*In-vitro* transformation of K5+/K19- and K5+/K19+ cells by mRas/mp53/mPIK3CA. A). K5^+^/K19^-^ or K5^+^/K19^+^ cell lines over-expressing vector or mRas/mp53/mPIK3CA oncogene combination were analyzed by Western Blotting. β-actin was used as a loading control. B). Representative images (magnification 20X) of colonies from K5^+^/K19^-^ and K5^+^/K19^+^ cells with vector or mRas/mp53/mPIK3CA oncogene combination assessed by anchorage independent growth assay. C) Quantification of colonies formed by different cells. Mean ± S.D. of a representative experiment done in triplicate is shown.

K5+/K19- and K5+/K19+ cell lines stably expressing mRas/mp53/mPIK3CA were generated. Western blotting shows transfected genes were expressed relatively similar levels in both cell lines (Fig 1A). To examine *in-vitro* transformation ability of K5+/K19- and K5+/K19+ transfectants, we performed anchorage independent growth assay. We observed that K5+/K19- and K5+/K19+ transfectants with oncogene combination mp53/mPIK3CA were able to form few small colonies in soft agar than their respective controls (S1 Fig). Notably, both K5+/K19- and K5+/K19+ transformed cells exhibited anchorage independent proliferation (Fig 1B) signifying an *in-vitro* transforming abilities of both cell lines and as observed previously (12) transformed K5+/K19+ cells formed more colonies than transformed K5+/K19- cells (Fig 1C).

### Oncogenic transformation induces complete EMT in K5+/K19- cells but not in K5+/K19+ cells

**Fig 2:**
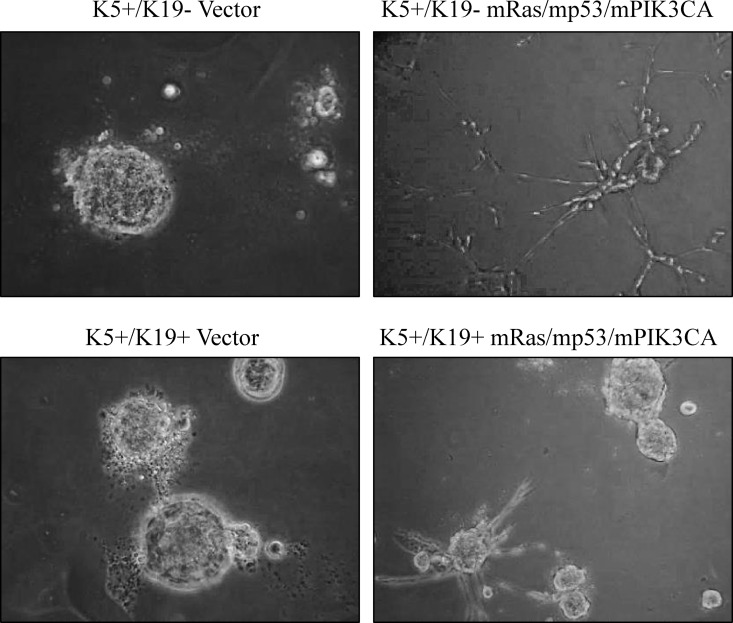
Differential EMT in mRas/mp53/mPIK3CA transformed K5+/K19- and K5+/K19+ cell lines. Representative phase contrast images of branched invasive structure and acini formed by vector or transformed K5^+^/K19^-^ or K5^+^/K19^+^ cells (magnification 20X).

EMT has been shown to induce stem cell expansion and metastatic behavior of cells [20]. However, recently it is becoming more apparent that mere up-regulation of EMT does not necessarily dictates the metastatic behavior of tumor cells [12, 21]. It is well documented that normal mammary epithelial cells produce round, polarized acinar structures in 3D matrigel, whereas transformed epithelial cells lose this ability and form irregular acinar structures in matrigel [22]. Notably, transformed K5+/K19- or K5+/K19+ cells exhibited different EMT phenotype. In this context, transformed K5+/K19- cells showed a complete EMT, whereas transformed K5+/K19+ cells had more epithelial phenotype with some cells with EMT appearance. To further examine the effect of transformation on their growth characteristic, we analyzed transformed K5+/K19- or K5+/K19+ cells for their ability to form polarized acinar structure in 3D-matrigel. EMT like transformed K5+/K19- cells lose their ability to form polarized acini and gave rise to branched invasive structures in matrigel (Fig 2). Whereas, transformed K5+/K19+ cells still maintain their ability to form acini and mostly produced rounded acini in matrigel with some branched invasive structure due to the partial EMT phenotype (Fig 2). These results show a differential effect of mRas/mp53/mPIK3CA oncogene combination on EMT induction in K5+/K19- or K5+/K19+ cells.

To exclude the possibility of clonal effect of EMT, we cloned K5+/K19- and K5+/K19+ cells and transformed an independent single clone from these two cell lines. Notably, again transformed K5+/K19- (clone 62) showed loose EMT-like colonies, while transformed K5+/K19+ (clone 21) showed epithelial phenotype (S2A Fig). These experiments clearly show cell type effect rather than clonal variation. Additionally, expression analysis of different EMT markers (E-Cadherin, vimentin) in these transformed cell lines showed expected decrease in E-Cadherin and increase in vimentin expression (S2B Fig)

### Oncogenic transformation of K5+/K19- or K5+/K19+ cells leads to increase in migration, invasion, as well as expansion of CD44^+^/CD24^low^ tumor initiating cell population

**Fig 3:**
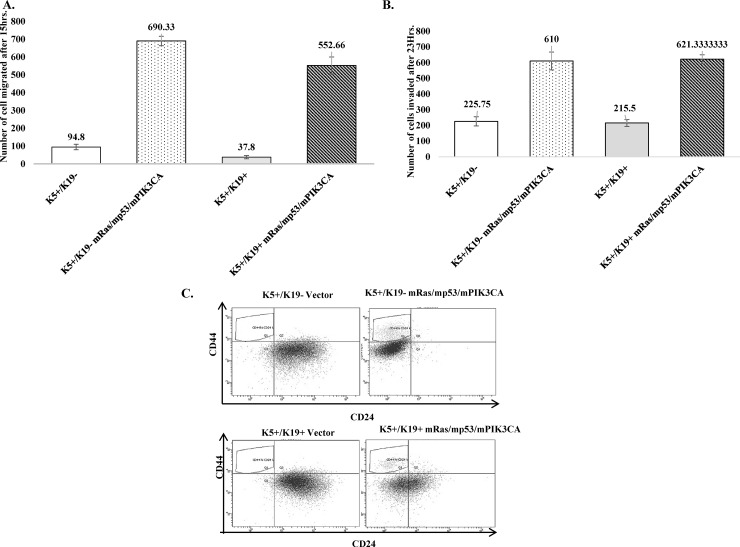
Transformed K5^+^/K19^-^ or K5^+^/K19^+^ cells exhibit increased ability to invade/migrate and showed increased proportion of tumor-initiating cells. (C) Vector or transformed K5^+^/K19^-^ or K5^+^/K19^+^ cells were stained with cell surface markers CD44 and CD24 and then analyzed by flow Cytometry. Dot plot analysis of cell population expressing CD44^+^/CD24^low^ (gated-upper left) is shown.

While anchorage independence and EMT are two important known phenotypic changes observed in cells upon transformation, it is now clear that invasion/migration and expansion of tumor initiating cells *in-vitro* also contribute to oncogenic behavior of cells *in-vivo*. Therefore, we first assessed migration and invasion abilities of the transformed cell lines *in-vitro*. Notably, transformed K5+/K19- as well as K5+/K19+ cells showed increased migration as well as invasion when compared to vector cells (Fig 3A and 3B). Notably, despite the differences in extent of EMT in transformed K5+/K19- or K5+/K19+ cells (Fig 2), their invasion or migration abilities were similar.

Next, we assessed expression of cell surface markers CD44 and CD24 in both transformed K5+/K19- and K5+/K19+ cells. CD44^+^/CD24^low^ is a marker for tumor-initiating cells [23] and its expression is associated with EMT like Claudin-low tumors [3]. We observed that K5+/K19- transformed cells that had undergone complete EMT *in-vitro* showed slightly more proportion of CD44^+^/CD24^low^ phenotype, when compared with transformed K5+/K19+ cells (5.1% vs. 3.7%) (Fig 3C). We also tested *in-vitro* self-renewal abilities of transformed cell lines by tumorsphere culture and found an increased self-renewal ability in different passages for both transformed K5+/K19- and K5+/K19+ cells (S3 Fig).These results are in accordance with the respective EMT morphologies observed for the two transformed cell lines and K5+/K19- transformed cells, as expected K5+/K19- cells showed higher CD44^+^/CD24^low^ population as compared to more epithelial like transformed K5+/K19+ cells.

### Transformed K5+/K19- and K5+/K19+ cells give rise to spindle like metaplastic carcinomas or adenocarcinomas, respectively upon *in-vivo* implantation

To confirm if the transformation ability observed *in-vitro* as a result of mRas/mp53/mPIK3CA over-expression in K5+/K19- and K5+/K19+ cells is also observed in *in-vivo* setting, we implanted the transformed cell lines in mammary gland of NSG mice. The vector cells, as expected from our previous published results did not show any tumor formation [12]. Significantly, both the transformed cell lines formed tumors in *in-vivo* setting. EMT like transformed K5+/K19- cells were more potent in forming tumors and all of the 4 mice formed primary tumors within 4 weeks of injection. Notably, transformed K5+/K19+ cells had a latency period slightly higher than transformed K5+/K19- cells where tumors were observed by 6^th^ week of injection (data not shown).

**Fig 4:**
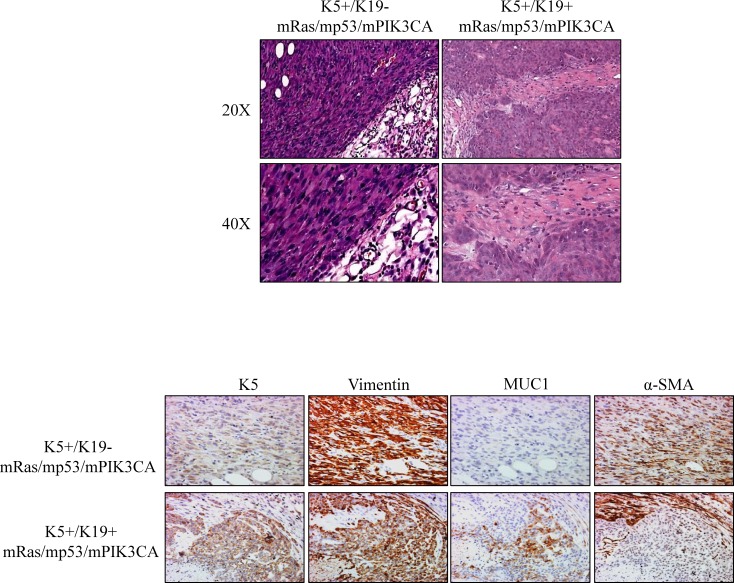
Transformed K5^+^/K19^-^ or K5^+^/K19^+^ cells produce in vivo tumors with distinct EMT characteristics. (A) Representative images of H&E staining of tumor sections (magnification 20X upper panel, 40X lower panel) from K5^+^/K19^-^ or K5^+^/K19^+^ cells over-expressing mRas/mp53/mPIK3CA. (B) Representative images of immunohistochemical staining of tumor sections derived from transformed K5^+^/K19^-^ or K5^+^/K19^+^ cells, with anti-CK5 (Basal/Stem), anti-vimentin (Basal/EMT), anti-MUC1 (Luminal) and anti-α-SMA (EMT) antibodies are shown.

**Fig 5:**
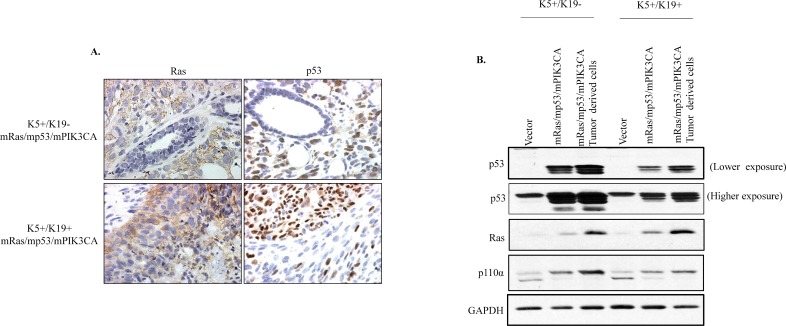
Expression of ectopically expressed genes in orthotropic tumors. **A.** Representative images of tumors from mp53/mRas/mPIK3CA transformed K5^+^/K19^-^ or K5^+^/K19^+^ cells immunostained with anti-Ras or anti-p53 antibodies. B. Western blot. Cell lysates from vector, transformed K5^+^/K19^-^ or K5^+^/K19^+^ cells or tumor cell lines derived from orthotopic tumors were probed with anti-Ras, anti-p53 and anti-PIK3CA(p-110α) antibodies, and then analyzed by Western Blotting. GAPDH was used as a loading control.

Notably, tumors formed by transformed K5+/K19- cells had a spindle like morphology and they resembled metaplastic carcinomas, whereas tumors derived from transformed K5+/K19+ resembled adenocarcinomas with a small spindle like metaplastic carcinoma componenent (Fig 4A). We then assessed the phenotypes of tumors by evaluating the expression of different makers for EMT, epithelial or stem-like cells such as MUC1 or E-Cadherin for epithelial, α-SMA or vimentin for EMT, and K5 or vimentin for stem or basal-like cells. Immunohistochemical staining of tumors from transformed K5+/K19- cells was in accordance with the EMT phenotype as observed in *in-vitro* cultures of these tumors, as they displayed α-SMA/Vimentin^+^/MUC1^-^ EMT-like phenotype (Fig 4B). Whereas, tumors derived from transformed K5+/K19+ cells exhibited mostly epithelial phenotype (α-SMA^+^/Vimentin^+^/MUC1^+^) with slight EMT phenotype (Fig 4B), consistent with their morphologies when cultured *in-vitro* prior to implantation. In order to verify that the tumors were indeed formed by the transformed K5+/K19- and K5+/K19+ cells, we assessed the protein expression of over-expressed oncogenes/tumor-suppresor (Ras, p53 and PIK3CA) in both orthotopic tumor as well as in tumor derived cell lines. Tumors from both cell types showed expected expression of transfected genes (Fig 5).

**Fig 6:**
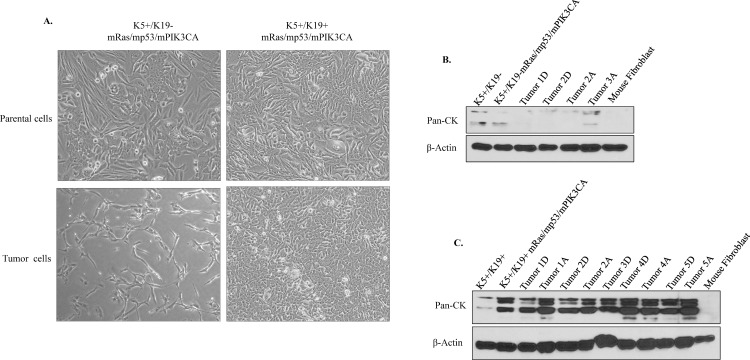
Tumor derived cells from transformed K5^+^/K19^-^ or K5^+^/K19^+^ maintain their EMT phenotype. Tumors formed by transformed K5^+^/K19^-^ or K5^+^/K19^+^ cells were excised and cultured to isolate primary tumor cells. (A) Phase contrast images of parental transformed K5^+^/K19^-^ or K5^+^/K19^+^ cells (upper panel) and tumor derived cells (lower panel) show differences in EMT phenotype between the K5^+^/K19^-^ or K5^+^/K19^+^ cells. (B) K5^+^/K19^-^ or (C) K5^+^/K19^+^ parental or tumor derived cell lines were analyzed by Western Blotting. β-actin was used as a loading control. A, refers to cells cultured in alpha-MEM medium and D, refers to DFCI medium.

Examination of tumor derived cells *in vitro* showed similar EMT or epithelial morphologies as observed in *in vivo* tumors (Fig 6A), suggesting that different phenotypes conferred by oncogene combination mRas/mp53/mPIK3CA is maintained by both the cell types regardless of microenvironment effect. EMT like tumor cells derived from primary tumors from transformed K5+/K19- cells had a drastically lower Pan-cytokeratin (CK) expression (Fig 6B). While tumors cells derived from primary tumors of transformed K5+/K19+ cells retained a high Pan-CK expression (Fig 6C) confirming their epithelial phenotype. Taken together, these results suggest that K5+/K19- cells overall have a higher susceptibilty to undergo EMT as compared to K5+/K19+ cells, suggesting EMT is mainly contributed by cell type of origin.

## Discussion

Heterogeneity of breast tumors is well known and is considered to be one of the causal factors for failure to therapeutic response in patients. Several studies have focused on understanding the etiology of breast cancer heterogeneity. Owing to several variables, such as the microenvironment changes, accumulation of mutations, loss of differentiation, it is rather difficult to understand the underlying factors those initiate or possibly mediate progression of the disease. In recent years, using defined cellular precursors and by introducing different oncogenes, we and others have shown that heterogeneity in breast tumors is likely to be caused by both the cell type, as well as alterations in genes [12, 24–26].

In the present study, we introduced mutant PIK3CA oncogene, which is known to be widely mutated in breast tumors, together with mutant Ras (widely used gene to *in vitro* transform mammary epithelial cells) and mutant p53 in two different stem/progenitor cell types K5+/K19- or K5+/K19+ and then assessed the effect of the oncogene combination in driving pathogenesis from the two isogenic cell lines. We observed that mRas/mp53/mPIK3CA combination was extremely potent in driving oncogenic phenotype *in-vitro* (Fig 1) as well as full tumorigenesis *in-vivo* (Fig 4). The most noticeable difference was transformed K5+/K19- cells exhibit high EMT characteristics and produced metaplastic like carcinomas, when compared to tumors those originated from transformed K5+/K19+ cells with same oncogene combination (Fig 4A and 4B). Furthermore, EMT was maintained in both *in-vitro* cells and in *in-vivo* tumors, as well as in tumor derived cell lines (Fig 6A). Additionally, we observed that mRas/mp53/mPIK3CA oncogene combination had a differential effect in driving EMT in K5+/K19- or K5+/K19+ cells, suggesting a cell type rather than oncogene type that determines EMT.

Interestingly, our previous observations on transformation of either cell types with oncogene combinations mRas/mp53/wtErbB2 or mRas/mp53/wtEGFRshowed EMT only in *in-vivo* tumors from K5+/K19- cells upon transformation with mRas/mp53/wtErbB2 combination of oncogenes [12]. Despite a complete EMT phenotype in tumors, K5+/K19- cells transformed with either mRas/mp53/wtErbB2 or mRas/mp53/wtEGFR had an epithelial morphology *in-vitro* [12]. Taken together, our previous and current study suggests that mutant PIK3CA is more potent inducer of EMT than wild-type ErbB2 or wild-type EGFR, and mutant PIK3CA can drive complete EMT in K5+/K19- cells.

Based on recent literature where mutant PIK3CA activates multipotency in different cell-types to generate heterogeneity of mice tumors [10], we analyzed the genes that are differentially regulated in the parental K5+/K19- and K5+/K19+ cells [12]. We found that, overall K5+/K19- cells have an up-regulated gene signature that corresponds to EMT like phenotype when compared with parental K5+/K19+ cells. THBS2, was the most highly expressed gene in K5+/K19- and this gene has been shown to be an important mediator of desmoplastic or stromal reaction in tumors [27]. The secreted protein activates the myofibroblasts and facilitates the development of niches that favor metastasis and likely cause metaplastic phenotypes in tumors [27]. Other genes including THBS2, COL5A1 and COL5A2, all of these were found to be highly expressed in K5+/K19- cells, form the metastasis activating or mesenchymal state inducing genes signature, as shown previously [28, 29]. Up-regulation of mesenchymal gene signature in K5+/K19- cellular precursors, may contribute to intrinsic susceptibilities of K5+/K19- cells to exhibit EMT.

It is however not necessary that induction of EMT itself governs the progression of disease. Our previous study and work from others, have shown that mere up-regulation of EMT gene signatures does not correlate with metastatic disease (Bhagirath et. al. manuscript in preparation) [12, 21, 30]. While EMT is important for migration of cells, success of metastatic colonization relies on reversion of this phenotype [27], and this capacity may vary from one cell to another. Consistently, although transformed K5+/K19- cells had a higher tendency to undergo EMT, their ability to form metastatic tumors was lower than transformed K5+/K19+ cells [12]. Our studies show that K19+ cells overall exhibit a higher transformation, as well as metastatic abilities [12]. This susceptibility of K19+ cells may reflect endogenous gene expression by the cell type [12]. Further investigation in to the gene expression changes in a K19+ cellular precursor, may help understand why these cells are more susceptible to metastasis.

## Conclusions

We demonstrate that ectopic over-expression of m-Ras/m-p53/mPIK3CA combination into K5+/K19- and K5+/K19+ stem/progenitor cells while transformed both cell types, only induced EMT in K5+/K19- cells, reflecting differential intrinsic susceptibilities of K19- or K19+ cellular precursors for EMT.

## Supporting Information

S1 Fig *In-vitro* transformation abilities of K5+/K19- and K5+/K19+ cells over-expressing mp53/mPIK3CA. A). Representative images (magnification 4X) of colonies from K5^+^/K19^-^ and K5^+^/K19^+^ cells with vector or mp53/mPIK3CA oncogene combination, as assessed by anchorage independent growth assay. B) Quantification of colonies formed by different cells. Mean ± S.D. of a representative experiment done in triplicate is shown.
(TIF)

S2 Fig Differential EMT in mRas/mp53/mPIK3CA transformed K5+/K19- and K5+/K19+ cell lines. (A) Phase contrast images showing differences in EMT phenotype in transformed K5^+^/K19^-^ (clone 62) or K5^+^/K19^+^ (clone 21) cells (magnification 10X). (B) Parental or transformed K5^+^/K19^-^ or K5^+^/K19^+^ cells were analyzed by western blotting for the expression of EMT markers- E-Cadherin or Vimentin. β-actin was used as a loading control.
(TIF)

S3 Fig *In-vitro* self-renewal abilities of transformed K5+/K19- or K5+/K19+ cell lines. A, B and C Quantification of tumorspheres formed by vector or mp53/mRas/mPIK3CA gene combination over-expressing K5^+^/K19^-^ and K5^+^/K19^+^ cells in different passages. Indicated cell lines were cultured in low-attachment plates in MEGM media for 3 weeks. Spheres ≥200μm were quantified.
(TIF)
