# Supplementary figures and images for: Mutant PIK3CA Induces EMT in a Cell Type Specific Manner
Source: PLoS One. 2016 Dec 12;11(12):e0167064. doi: 10.1371/journal.pone.0167064 (PMC5152840; doi:10.1371/journal.pone.0167064)

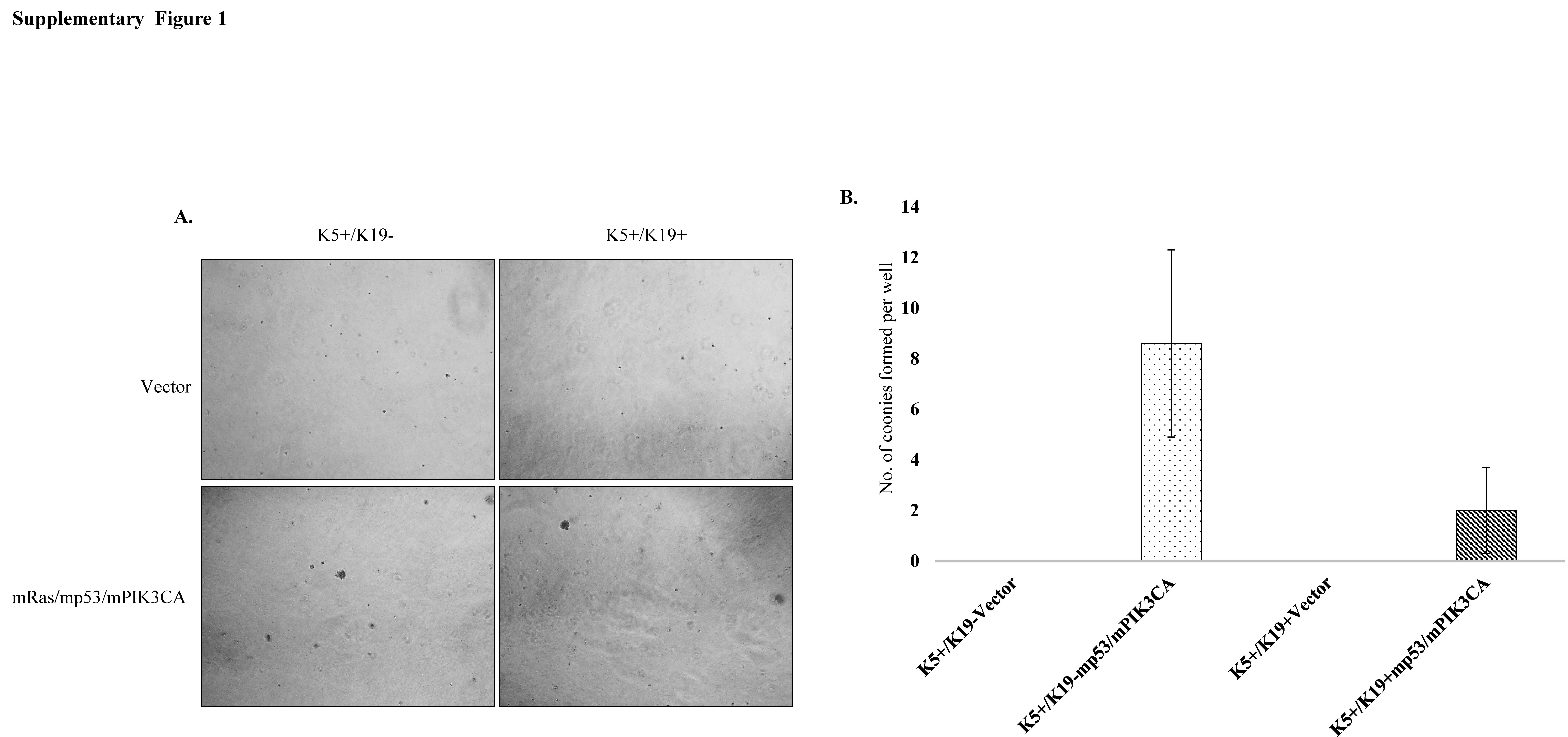

Supplement: S1 Fig — A). Representative images (magnification 4X) of colonies from K5+/K19- and K5+/K19+ cells with vector or mp53/mPIK3CA oncogene combination, as assessed by anchorage independent growth assay. B) Quantification of colonies formed by different cells. Mean ± S.D. of a representative experiment done in triplicate is shown. (TIF) [file pone.0167064.s001.tif]

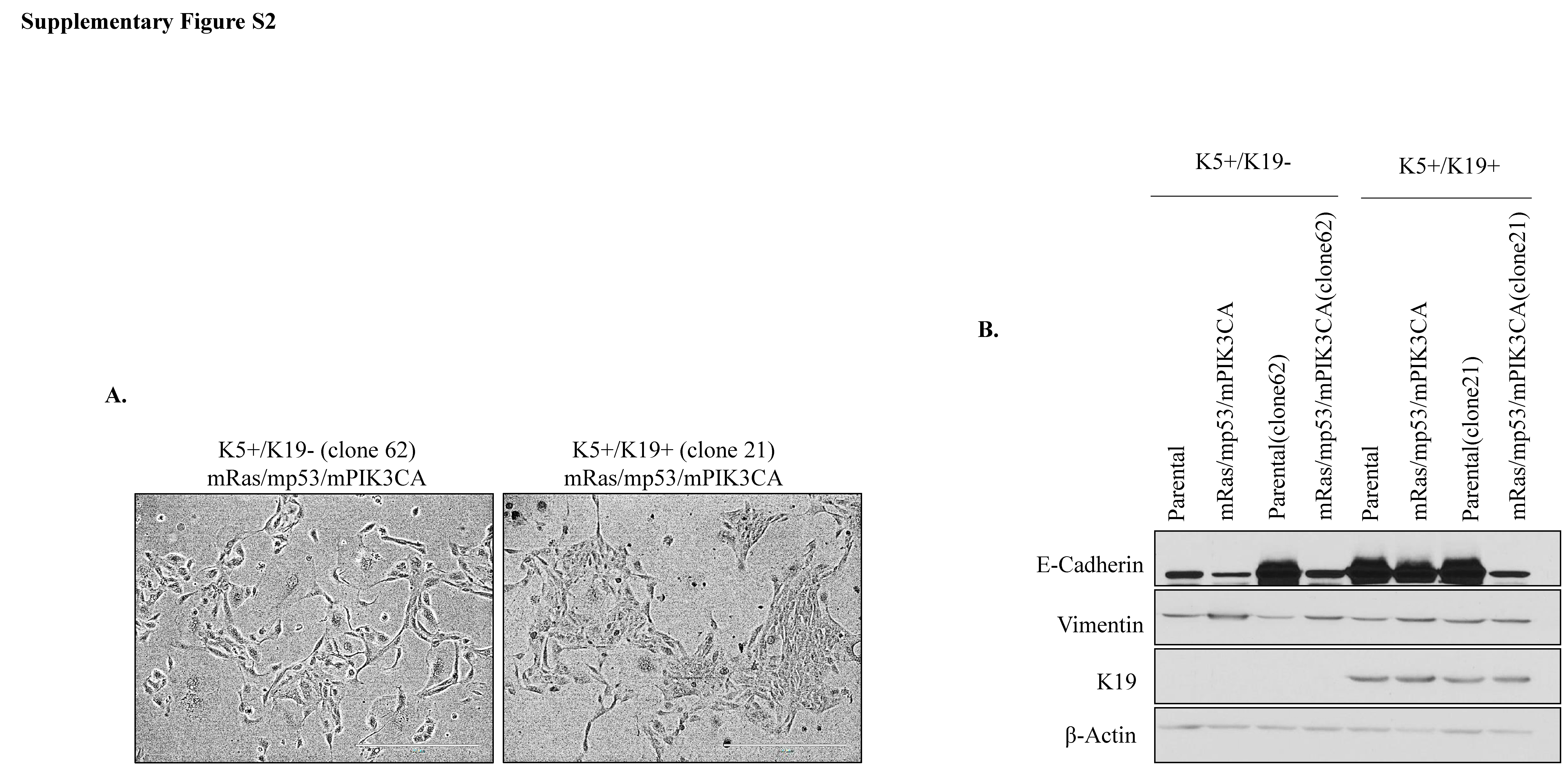

Supplement: S2 Fig — (A) Phase contrast images showing differences in EMT phenotype in transformed K5+/K19- (clone 62) or K5+/K19+ (clone 21) cells (magnification 10X). (B) Parental or transformed K5+/K19- or K5+/K19+ cells were analyzed by western blotting for the expression of EMT markers- E-Cadherin or Vimentin. β-actin was used as a loading control. (TIF) [file pone.0167064.s002.tif]

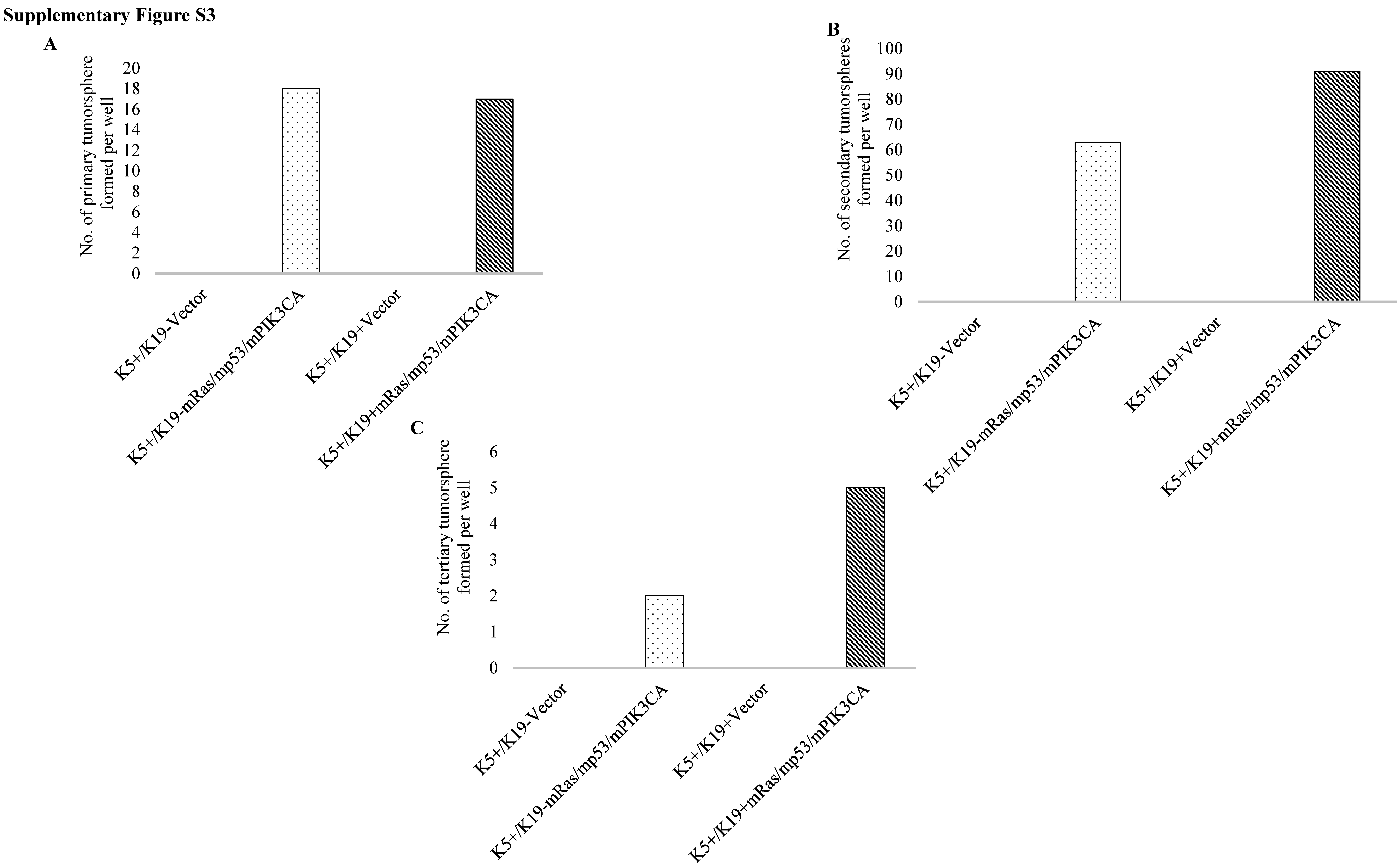

Supplement: S3 Fig — A, B and C Quantification of tumorspheres formed by vector or mp53/mRas/mPIK3CA gene combination over-expressing K5+/K19- and K5+/K19+ cells in different passages. Indicated cell lines were cultured in low-attachment plates in MEGM media for 3 weeks. Spheres ≥200μm were quantified. (TIF) [file pone.0167064.s003.tif]
